# Proteomic Characterization of Urinary Extracellular Vesicles from Kidney-Transplanted Patients Treated with Calcineurin Inhibitors

**DOI:** 10.3390/ijms21207569

**Published:** 2020-10-14

**Authors:** Laura Carreras-Planella, Javier Juega, Omar Taco, Laura Cañas, Marcella Franquesa, Ricardo Lauzurica, Francesc Enric Borràs

**Affiliations:** 1 REMAR-IVECAT Group, Can Ruti Campus, Germans Trias i Pujol Health Science Research Institute, 08916 Badalona, Spain; lcarreras@igtp.cat (L.C.-P.); juega.javier@gmail.com (J.J.); laucanyas78@hotmail.com (L.C.); mfranquesa@igtp.cat (M.F.); rlauzurica.germanstrias@gencat.cat (R.L.); 2 Department of Cell Biology, Physiology and Immunology, Autonomous University of Barcelona, Bellaterra, 08193 Cerdanyola del Vallès, Spain; 3 Autonomous University of Barcelona, Bellaterra, 08193 Cerdanyola del Vallès, Spain; oetaco.germanstrias@gencat.cat; 4 Nephrology Department, Can Ruti Campus, Germans Trias i Pujol University Hospital, 08916 Badalona, Spain; 5 Instituto de Salud Carlos III, Red de Investigación Renal (ISCIII-REDinREN RD16/0009 Feder Funds), 28029 Madrid, Spain

**Keywords:** exosomes, renal transplantation, tacrolimus, cyclosporine A, proteomics

## Abstract

Use of immunosuppressive drugs is still unavoidable in kidney-transplanted patients. Since their discovery, calcineurin inhibitors (CNI) have been considered the first-line immunosuppressive agents, in spite of their known nephrotoxicity. Chronic CNI toxicity (CNIT) may lead to kidney fibrosis, a threatening scenario for graft survival. However, there is still controversy regarding CNIT diagnosis, monitoring and therapeutic management, and their specific effects at the molecular level are not fully known. Aiming to better characterize CNIT patients, in the present study, we collected urine from kidney-transplanted patients treated with CNI who (i) had a normal kidney function, (ii) suffered CNIT, or (iii) presented interstitial fibrosis and tubular atrophy (IFTA). Urinary extracellular vesicles (uEV) were enriched and the proteome was analyzed to get insight into changes happening during CNI. Members of the uroplakin and plakin families were significantly upregulated in the CNIT group, suggesting an important role in CNIT processes. Although biomarkers cannot be asserted from this single pilot study, our results evidence the potential of uEV as a source of non-invasive protein biomarkers for a better detection and monitoring of this renal alteration in kidney-transplanted patients.

## 1. Introduction

The calcineurin inhibitors (CNI) cyclosporine A [1,2] and tacrolimus have become the current first-line immunosuppressive agents in kidney transplantation [3,4,5] for more than 30 years. Their main mechanism of action is based on the disruption of T-cell activation and proliferation by inhibiting calcineurin, the enzyme responsible of the dephosphorylation and activation of NFATc that triggers the transcription of interleukin (IL)-2. Hampering IL-2/IL2R interaction reduces T-cell activation and proliferation, a crucial step in graft rejection [6]. About 94% of renal-transplanted patients receive a CNI-based immunosuppressive regime [7], yet it is well-known that CNI treatment can produce nephrotoxicity, commonly referred to as CNI toxicity (CNIT) [8]. There is still controversy in the diagnosis, monitoring, and therapeutic management of CNIT (reviewed in [9]). Acute CNIT (aCNIT) is histologically associated with the appearance of isometric vacuolization of the tubular epithelium [10,11,12], while chronic CNI (cCNIT) produces chronic renal lesions such as interstitial fibrosis, tubular atrophy, arteriolar hyalinosis, and glomerulosclerosis [13,14], all contributing to the progressive and irreversible deterioration of the renal function and graft-loss [15,16]. To avoid this scenario, CNI levels in patients should be maintained within a narrow therapeutic window, a big challenge due to the high inter- and intraindividual pharmacokinetic variability of these drugs [17,18]. There are currently no specific markers of CNIT, and tacrolimus or cyclosporine A trough levels not always correlate with CNIT [19].

Urinary extracellular vesicles (uEV) have been shown to carry proteins that reflect the pathophysiological state of cells in the urinary system [20]. Thus, the analysis of uEV can shed light on the pathophysiological processes occurring in the kidney and as well as providing a source of non-invasive biomarkers of renal alterations. With the aim of characterizing CNIT in renal transplantation, we collected urine samples and isolated uEV from kidney-transplanted patients (all treated with CNI) that were classified into: (i) normal kidney function (NKF), (ii) CNIT, or (iii) interstitial fibrosis and tubular atrophy (IFTA). The uEV proteome was characterized by mass-spectrometry.

## 2. Results

### 2.1. Patients and Samples

In this study, size-exclusion chromatography (SEC)-isolated uEV samples from three groups of kidney-transplanted patients (NKF, IFTA, and CNIT) were used. Table 1 summarizes the clinical data of each patient when urine sample was collected. As expected, patients in the NKF group presented significantly lower serum creatinine levels than IFTA (*p* = 0.012) and CNIT (*p* = 0.012), but no other significant differences were found. Patient C10, the unique patient affected by chronic CNIT, presented the highest serum creatinine level. Table 2 summarizes the induction treatment at kidney transplantation, immunosuppression regime at sample’s collection and the diagnosis based on renal biopsy and clinical parameters. All patients were receiving an immunosuppressive regime consisting of prednisone and a calcineurin inhibitor (in most cases tacrolimus, only one patient in each group was receiving cyclosporine A), with or without mycophenolate mofetil. The histopathological results of the Banff scoring are summarized in Supplementary Table S1 and representative histological photographs are displayed in Supplementary Figure S1. Acute CNIT was diagnosed in four out of five cases by the presence of isometric vacuolization of the tubular epithelium and the preservation of the microvilli on the apical border. The other CNIT patient was diagnosed with chronic CNIT because of the presence of grade 3 arteriolar hyalinosis and circumferential hyalinosis with peripheric nodules. The diagnosis of CNIT was further supported by the high blood levels of tacrolimus, determined according to the study by Cosio et al. [21] or high blood levels of cyclosporine A based on the Symphony study [22]. Patients in the IFTA group presented different grades of fibrosis in the renal biopsy with no other signs of pathology. The determination of IFTA grade was based on the mean values of the Banff parameters chronic interstitial and tubular lesions (ci and ct). Also, IFTA patients showed lower blood levels of tacrolimus and cyclosporine A compared to CNIT patients, and similar to NFK patients. Patient I13 suffered a previous episode of acute cellular rejection and one episode of acute humoral rejection 21 and 9 months before urine collection, respectively. This patient showed no histopathological signs of rejection at sample collection and was therefore included in the study.

### 2.2. Global Analysis of the uEV Proteome

A total of 730 proteins were confidently identified after processing mass spectrometry data. Confirming previous results, and as expected by the enrichment technique used, the FunRich analysis of the identified proteins revealed that the most significantly enriched terms were those related to the secretion of EV such as “vesicle mediated transport” or “extracellular region” according to Gene Ontology (GO)—Biological Process (BP) and Cellular Component (CC) enrichment analysis, respectively (Supplementary Tables S2 and S3).

**Figure 1:**
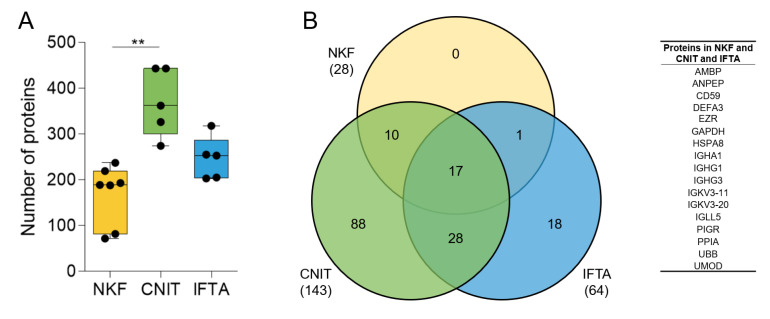
(**A**) Number of proteins found by mass-spectrometry in uEV samples in each group. Whiskers represent minimum to maximum; horizontal line represents the mean (** *p* < 0.01). (**B**) Venn diagram showing the number of coinciding proteins between the samples of each group (in brackets) and between the all the samples in the study (number in the corresponding circles). On the right, list of the 17 proteins found in all samples.

The number of identified proteins in CNIT samples (mean ± sd, 369 ± 73.9) was significantly higher compared to NKF samples (168.6 ± 65.1) and higher than IFTA samples (246.8 ± 47.0) (Figure 1A).

We then assessed the homogeneity of the samples within each group. First, the number of shared proteins among the samples in each group with respect to the total number of proteins identified in the group was analyzed. The seven NKF samples shared up to 28 proteins of a total of the 394 in the group (7.1%). Five CNIT patients shared up to 143 of 621 proteins (23.0%), and five IFTA patients shared 64 of 512 proteins (12.5%). In total, 17 proteins were shared among all samples analyzed (Figure 1B).

**Figure 2:**
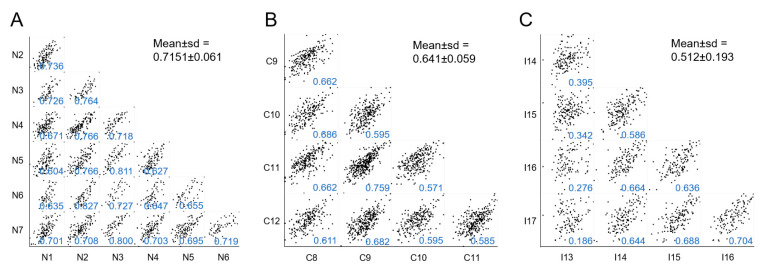
Multi-scatter plots showing correlations of samples within each group: (**A**), NKF; (**B**), CNIT; and (**C**), IFTA. In each individual plot the Pearson correlation coefficients are shown in blue and the corresponding mean ± sd for each group is shown in black.

Second, we performed a multiple correlation analysis among samples included in each group as a measure of intragroup homogeneity. Each sample’s protein expression was compared with every other sample in the same group to obtain the mean of all Pearson correlation coefficient. NKF and CNIT groups were the most homogeneous (mean Pearson coefficient > 0.6) (Figure 2A,B). Conversely, the IFTA group showed a lower level of internal homogeneity (barely > 0.5) (Figure 2C). In this group, sample I13 presented a low Pearson coefficient when individually tested with every other IFTA sample (Pearson coefficients < 0.400), suggesting a particular behavior, as observed later. Of note, if I13 sample was not considered in this assay, the mean Pearson coefficient of IFTA samples increased to 0.654, a value similar to that obtained in the CNIT group.

### 2.3. Differentially Expressed Proteins

**Figure 3:**
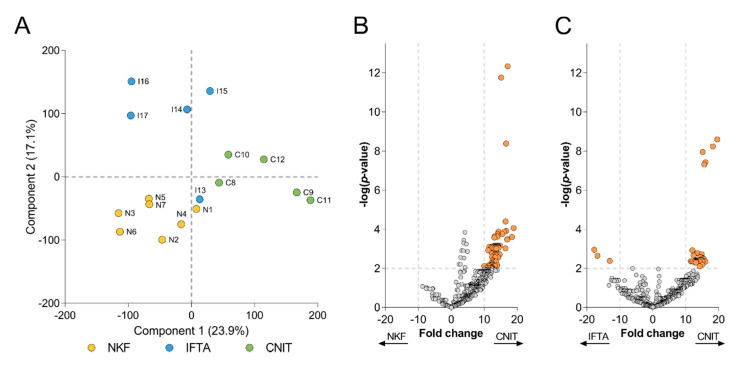
(**A**) Principal component analysis (PCA) biplot that shows distribution of samples according to Components 1 and 2. Each circle represents a sample, which are labelled and colored according to their group. Volcano plots depict the differentially expressed proteins (**B**) between CNIT and NKF, and (**C**) between CNIT and IFTA. Each circle represents a protein. On y-axis −log(*p*-value) from a t-test is represented, with a dashed line at *p* < 0.01 to indicate significance, over which proteins are colored in orange. The expression fold change is represented on the x-axis, with dashed lines at >10 and <−10.

A principal component analysis (PCA) was performed in order to get more insight onto the global protein variation in the two renal alterations (CNIT and IFTA) and the NKF groups (Figure 3A). CNIT patients were clearly segregated from IFTA and NKF patients by Component 1, which accounted for a 23.9% of the variability among samples, while Component 2, which accounted for 17.1% of the variability, permitted to segregate the three groups of samples. Only the sample of the IFTA group (I13), which had a low correlation with the other IFTA samples, did not cluster together with the rest of samples in its group.

Based on this observation, a more concise comparison was performed using a volcano plot to depict the proteins that were significantly overexpressed between groups. Those proteins having *p* < 0.01 and fold change >10 or <−10 in each comparison were considered as more relevant. From 71 proteins found more expressed in CNIT samples compared to NKF samples (Figure 3B), three (CTSZ, RAB8A and SERPINC1) showed a notable low *p*-value (<10^−8^). On the other hand, up to 39 proteins were significantly more expressed in the CNIT group compared to IFTA patients (Figure 3C), among which five proteins (ADIRF, CAPG, STXBP2, GNAI1, and ATP1A1) presented a remarkably lower p-value (<10^−7^) Of note, no proteins were overexpressed in the NKF group and only three proteins (HIST1H4A, HRG, and IGHV4-28) were significantly more expressed in the IFTA group versus CNIT. The full list of significant proteins from the volcano plots can be seen in Supplementary Tables S4 and S5.

### 2.4. Biological Processes Enrichment Analysis

After identifying significant proteins differentially expressed in CNIT, the GSEA software was used to reveal the GO biological processes that were up- or downregulated in this group compared to the other groups (each gene being equivalent to a protein). A total of 45 gene sets were significantly enriched (nominal *p*-value < 0.05) in CNIT compared to NKF. None of them reached the minimal significant false discovery rate (FDR) of 0.25, probably because of the dimension of the difference in protein numbers and level of expression. Nevertheless, the most overexpressed gene set was “Negative regulation of immune response” (Supplementary Tables S6 and S7).

**Figure 4:**
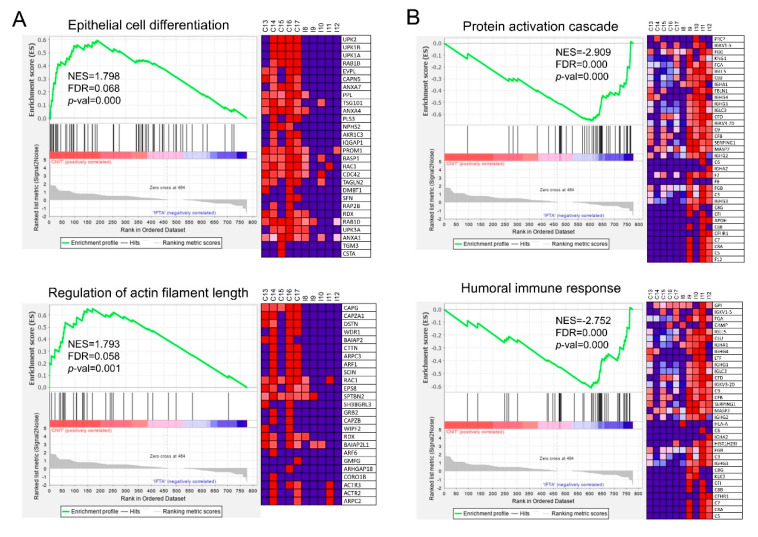
Enrichment plots from GSEA conducted with GO-BP gene sets, each gene accounting for one protein. Statistically significant up-regulation of (**A**) “epithelial cell differentiation” and “regulation of actin filament length” was found in CNIT when compared with IFTA (to the left of the x-axis, positive running enrichment score (ES)). (**B**) “Protein activation cascade” and “humoral immune response” were found up-regulated in IFTA compared to CNIT (to the right of the x-axis, negative ES). Vertical black lines indicate the position of individual genes of the gene set in the ranked list. Heatmap on the right of each plot show the relative expression level of the most up-regulated genes of the gene set (red = high, blue = low). NES, normalized enrichment score; FDR, false discovery rate; *p*-val, *p*-value.

When comparing CNIT and IFTA, up to 128 gene sets were significantly upregulated (FDR < 0.25) in CNIT patients (Supplementary Table S8). The most overexpressed gene sets were “epithelial cell differentiation” and “regulation of actin filament length” (Figure 4A). In addition, CNIT presented overexpression of vesicle-related gene sets such as “vesicle organization” or “multivesicular body organization.” Other gene sets more expressed in CNIT than in IFTA were “cell cycle” and “intracellular protein transport.”

On the reverse comparison, 59 gene sets were upregulated in IFTA compared to CNIT (Supplementary Table S9), “protein activation cascade” and “humoral immune response” (Figure 4B) being two of the most significant ones. Other gene sets related to inflammatory response and complement activation were also upregulated.

**Figure 5:**
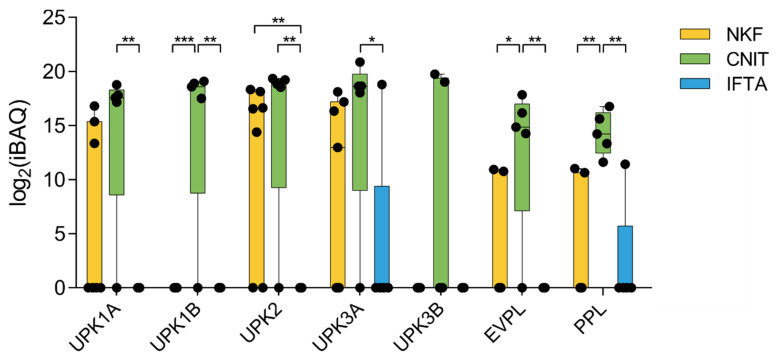
Expression profile of seven proteins of the uroplakin and plakin families. Whiskers represent minimum to maximum; horizontal line represents the mean. UPK1A, uroplakin 1A; UPK1B, uroplakin 1B; UPK2, uroplakin 2; UPK3A, uroplakin 3A; UPK3B, uroplakin 3B; EVPL, envoplakin; PPL, periplakin. * *p* < 0.05; ** *p* < 0.01; *** *p* < 0.001.

Interestingly, several proteins of the uroplakin family (UPK1A, UPK1B, UPK2, and UPK3A), as well as envoplakin (EVPL) and periplakin (PPL) (citolinker proteins) were significantly upregulated in CNIT compared to IFTA and NKF (Figure 5). These proteins are members of the “epithelial cell differentiation” gene set.

## 3. Discussion

CNIT in kidney-transplantation is controversial. In this study, the uEV proteome of kidney-transplanted patients diagnosed with CNIT was analyzed and compared to either kidney-transplanted patients with clinically normal kidney function or diagnosed with IFTA without CNIT, all of them receiving a similar immunosuppressive regime including CNI. Expectedly, both CNIT and IFTA patients presented a significantly higher serum creatinine than NKF patients.

A first characterization of all the proteins found by mass-spectrometry showed that there was an enrichment of proteins related to the secretion of EV, which denotes the efficacy of uEV enrichment performed using SEC. An efficient EV purification is key to greatly diminish the interference of abundant soluble proteins (especially uromodulin) for a mass-spectrometry analysis, contributing to the detection of lower abundance proteins that may be potential biomarkers [23,24,25].

The PCA-based comparison of the proteomic results could clearly separate the three groups of samples, indicating that the uEV proteome follows different patterns in NKF, CNIT, and IFTA. Only sample I13, which showed a low correlation coefficient with the other samples within its group, did not cluster as the other IFTA samples did. A possible explanation for this observation could be the two previous episodes of rejection that I13 patient had suffered within 2 years before sample collection. Yet, as no signs of rejection were observed in the biopsy performed at the time of urine sample collection, the patient was finally included in the assay. Our results may suggest those rejection episodes do seem to be still reflected in the uEV proteome. Also interestingly, although chronic CNIT can present lesions compatible with an IFTA diagnosis at the histological level [26], sample C10 did not cluster with IFTA samples. In fact, despite being the unique CNIT sample diagnosed of a chronic CNIT instead of acute CNIT, there was no apparent segregation of C10 from the other CNIT samples in the PCA, pointing to the resemblance of the pathological process in both chronic and acute cases, at least at the uEV proteomic level. Yet, as this study has been performed on a limited number of samples, these results have to be cautiously interpreted.

Vesicle-related gene sets were significantly overexpressed in the CNIT group. Some studies have described that acute CNIT can cause tubular epithelial cell cytoplasmic small vacuoles and abundant lysosomes due to dilatations of the smooth endoplasmic reticulum by aqueous fluid [10,11,12]. Moreover, it has been shown that a pathological process, like CNIT, increases the secretion activity of kidney cells [27]. Hypothetically, some of these vesicles may be released into the lumen of the proximal tubules and then in urine, so they would be captured as extracellular vesicles, thus increasing the number of proteins found in the proteomic analysis in CNIT samples, as we report here.

In renal biopsies, CNIT often show features shared with IFTA lesions. Both cyclosporin-A and tacrolimus are directly responsible of the increase of TGF-β1 [28], a factor that promotes interstitial fibrosis by increasing synthesis of proteins of the extracellular matrix and decreasing their degradation [29,30]. Moreover, TGF-β1 induces epithelium-to-mesenchymal transition at the tubular level leading to fibrosis by the generation of myofibroblasts [31,32]. Also, it has been shown that CNI drugs induce apoptosis on tubular and interstitial cells in vitro [33,34]. The GSEA analysis show overexpression of different gene sets in CNIT compared to IFTA as well as NKF, suggesting the activation of specific mechanisms in CNIT. Specifically, the proteome of CNIT was significantly enriched in gene sets related to epithelial cell differentiation, probably because of the death of tubular epithelial cells that force their regeneration. Members of the uroplakin family (UPK1A, UPK1B, UPK2, and UPK3A) were also overexpressed in CNIT compared to IFTA. Uroplakins are transmembrane proteins that bind to each other to form a plaque on the surface of the urothelium to prevent influx of urine from the lumen, which covers the renal pelvis, ureters, urinary bladder, and prostatic urethra [35]. The molecular weight of uroplakins ranges from 15 to 47 kDa, suggesting their intravesicular location [36]. Periplakin and envoplakin, two other members of the plakin family that function as cell-linker proteins, were also found enriched in CNIT [37]. These two proteins present a larger molecular weight of around 200 kDa and would possibly elute in the uEV-enriched fractions of SEC as free proteins instead of being carried by uEV [38,39]. Salih et al. described that patients in advanced stages of autosomal dominant polycystic kidney disease (ADPKD) presented increased levels of periplakin and envoplakin in their uEV [40]. In our study, only one patient in the CNIT group (C11) was diagnosed with ADPKD, but all the other CNIT samples also presented high levels of periplakin and envoplakin. The higher abundance of plakins in CNIT uEV suggests that the toxic effect of CNI on the urothelium may increase citolinker proteins’ activity and this would be reflected in a higher presence in their uEV. Therefore, plakin family members could represent promising biomarkers for CNIT in uEV.

Conversely, the CNIT uEV proteome did not reflect genes related to protein activation cascade and humoral response as well as other inflammatory processes when compared to IFTA. The reason behind could be that patients in the CNIT group had higher serum levels of CNI [21] which could be responsible for an increased capacity to suppress the inflammatory response.

Only a few studies using proteomic approaches have defined the effect of CNIT on kidneys. Sidgel et al. [41] investigated the urinary proteome of kidney-transplanted pediatric patients with different pathologies, including CNIT. They found a panel of ten proteins that potentially differentiated CNIT from chronic allograft nephropathy, although none of them coincided with our results, most probably because of the differences between using whole urine or uEV. Other groups studied the effect of CNI on renal cell lines in vitro [42,43]. They found that CNIT caused an increase in endoplasmic reticulum stress, mitochondrial apoptosis, and involvement of the phosphatidylinositol 3-kinases (PI3K), metalloproteinases (MMP), protein kinase C (PKC), and glycogen synthase kinase 3 (GSK3) pathways. To our best knowledge, our study is the first to analyze the uEV proteome of kidney-transplanted patients diagnosed of CNIT.

There is a lack of knowledge on how CNIT develops in renal transplanted patients despite the wide use of CNI and their contribution to kidney graft loss. Thus, a better knowledge of the effect of CNI at the renal level is of utmost importance for the detection and management in patients undertaking kidney transplantation. Although no conclusive biomarkers can be asserted from this single pilot study, we found a higher expression of proteins from the plakin family in the CNIT group, which may be envisaged as promising biomarkers and merit future investigation. This work adds further evidence to the potential of uEV as a source of non-invasive protein biomarkers for the better detection and monitoring of this renal alteration in kidney-transplanted patients.

## 4. Materials and Methods

### 4.1. Patients and Pre-Processing of Samples

Urine samples were collected from kidney-transplanted patients classified into three groups: normal kidney function (NKF) (*n* = 7) as determined by analytical parameters (creatinemia and proteinuria), CNIT (*n* = 5), and interstitial fibrosis tubular atrophy (IFTA) (*n* = 5), both diagnosed by analytical parameters and by per-cause renal biopsy. Patients classified in the IFTA group had a biopsy diagnosis of IFTA in the absence of any other cause. None of the patients presented C4d deposits in the renal biopsy. Morning mid-stream urine was collected immediately before per-cause renal biopsy (in the case of alteration of serum creatinine and proteinuria). Inclusion criteria were male or female patients older than 18 years of age and ability to give informed consent. Exclusion criteria were hematuria or urinary tract infection demonstrated by the presence of leukocyturia and/or bacteriuria and a previous kidney transplantation. All patients were receiving a similar immunosuppressive regime including CNI. This study was performed in line with the principles of the Declaration of Helsinki [44]. Approval was granted by the Ethics Committee “Comitè d’Ètica de la investigació clínica de l’Hospital Universitari Germans Trias i Pujol” and in accordance with its recommendations of the Guideline for Good Clinical Practice. Informed consent was obtained from all individual participants included in the study. An arbitrary number was used to label samples to protect patients’ identity.

### 4.2. Samples Processing and uEV Isolation

Urine samples were centrifuged (600× *g* 15 min) to eliminate cells and debris and stored at −80 °C with protease inhibitor (AEBSF, 0.138 mg/mL; Roche, Basel, Switzerland). Urinary EV (uEV) were isolated following the procedure described by Monguió-Tortajada et al. [45]. In brief, urine samples (140 mL) were thawed overnight at 4 ºC and centrifuged at 17,000× *g* for 10 min. The pellet was treated with 1,4-dithiothreitol (200 mg/mL; Sigma-Aldrich, St. Louis, MO, USA) to disrupt Tamm-horse fall protein polymers before mixing it with the initial supernatant and centrifuging again at 17,000× *g* for 10 min. Then, the supernatant was concentrated through a Centricon filter unit of 100 kDa cut-off (Millipore, Bedford, MA, USA) down to 1–2 mL. One mL of the concentrate was loaded onto a SEC column with 12 mL of sepharose CL-2B (Sigma-Aldrich) and 20 fractions of 500 µL were collected using phosphate-buffered saline (PBS; Sigma-Aldrich) as elution agent. Protein elution of SEC fractions was determined by reading absorbance at 280 nm with Nanodrop ND-1000 (Thermo Scientific, Waltham, MA, USA). In addition, SEC fractions were analyzed for their CD9 and CD63 content (typical tetraspanin EV markers) using beads-based assay flow cytometry. The three fractions with the highest CD9 and CD63 mean fluorescence intensity (MFI) intensity were pooled together rendering a volume of approximately 1.5 mL of uEV-enriched samples.

### 4.3. Mass-Spectrometry

Five hundred µL of uEV-enriched samples were used for the mass-spectrometry analysis (LC-MS/MS) on an Orbitrap XL (Thermo Scientific). Proteins were digested using LysC and Trypsin (Promega, Madison, WI, USA) and BSA (Sigma-Aldrich) solutions were included as quality controls. Data were analyzed using the Proteome Discoverer software (v2.0; Thermo Scientific) and proteins were identified using Mascot (Matrix Science, London, UK) against the SwissProt human database (UniProt, April 2015; https://www.uniprot.org/) [46] with an FDR of 0.05. The resulting raw data files were processed using the MaxQuant software [47] (version 1.5.3.30) against the SwissProt human database (UniProt, December 2015) applying a maximum FDR of 1%. Intensity-based absolute quantification (iBAQ) values normalized with the EV marker ezrin were used for subsequent analyses.

### 4.4. Proteomics and Statistical Analysis

The FunRich software (http://www.funrich.org, Melbourne, Australia) [48,49] was used to perform some of the GO enrichment analyses. Overlapping proteins were calculated and visualized with the online tool InteractiVenn (http://www.interactivenn.net/) [50]. The Perseus software [51] (v1.5.6.0; http://www.perseus-framework.org, Max Planck Institute of Biochemistry, Martinsried, Germany) and GraphPad Prism software (v6.0 GraphPad Software, San Diego, CA, USA) were used for the creation of plots and the statistical analysis. After testing for normality, two-sided unpaired *t*-test (parametric) or Mann-Whitney test (non-parametric) were used for the comparison of two groups of samples; in the case of multiple-groups comparison, one way ANOVA with Holm-Sidak’s multiple comparison test (parametric) or Kruskall-Wallis with Dunn’s multiple comparison test (non-parametric) were performed. Finally, the Gene Set Enrichment Analysis software (GSEA v3.0, Broad Institute, Cambridge, MA, USA) [52] was used to compare the enrichment of gene sets, which were downloaded from the GSEA Molecular Signatures Database (MSigDB v6.2, Broad Institute, Cambridge, MA, USA) [53]. The normalized enrichment score (NES) accounts for differences between gene sets to allow comparisons between them. The FDR represents the nominal *p*-value adjusted for gene set size and multiple hypothesis testing. It is the estimated probability that a gene set with a given NES represents a false positive finding (significant FDR < 0.25, as recommended by the GSEA software).

## 5. Patents

LCP, RL and FEB have a European Patent Application pending for the present work.

## Figures and Tables

**Table 1:** Clinical parameters of the study patients at urine collection.

Group, Sample	Age	Gender	DM	HT	Crea.	Prot.	Months from RT	LD	Donor Age
N1	64	F	−	−	0.86	99	137.9	−	53
N2	65	M	−	+	0.86	110	186.6	−	37
N3	45	F	−	+	0.82	30	113.6	−	43
N4	42	M	−	+	0.89	187	186.6	−	30
N5	57	M	−	+	0.9	92	166.4	−	45
N6	65	F	−	−	0.85	55	70.0	+	37
N7	69	F	−	+	1.14	86	57.8	+	58
C8	55	M	+	+	2.29	506	0.5	+	62
C9	33	M	−	−	1.93	232	2.8	+	59
C10	49	F	−	+	3.08	427	238.8	−	45
C11	50	F	−	−	2.49	207	5.5	−	60
C12	41	F	−	+	1.80	76	25.7	−	34
I13	50	F	−	−	2.00	62	21.7	+	48
I14	64	M	−	+	1.60	1600	84.6	−	71
I15	68	M	−	+	2.49	94	25.8	−	67
I16	68	M	+	+	2.62	800	15.1	−	38
I17	53	F	−	+	2.30	806	252.3	−	35
Sig.	ns	ns	ns	ns	***	ns	ns	ns	ns
*p*-value	0.064 ^a^	0.784 ^b^	0.452 ^b^	0.784 ^b^	<0.001 ^a^	0.093 ^a^	0.113 ^a^	0.784 ^b^	0.387 ^a^

^a^ Kruskall–Wallis test or ^b^ Chi-squared test were performed to determine statistical differences between groups. N, NKF, C, CNIT; I, IFTA; DM, diabetes mellitus type 2; HT, arterial hypertension; Crea., serum creatinine (mg/dL); Prot., proteinuria (mg/g creatinine); Months from RT., months from renal transplantation until sample collection; LD, living donor; F, female; M, male.

**Table 2:** Immunosuppression regime at urine collection.

Group, Sample	Induction Treatment	IS at Urine Collection	High CNI	Diagnosis
N1	IL2RA	PR, CSA	−	NKF
N2	rATG	PR, TAC, MMF	−	NKF
N3	IL2RA	PR, TAC	−	NKF
N4	IL2RA	PR, TAC	−	NKF
N5	IL2RA	PR, TAC	−	NKF
N6	IL2RA	PR, TAC, MMF	−	NKF
N7	IL2RA	PR, TAC, MMF	−	NKF
C8	IL2RA	PR, TAC, MMF	+	aCNIT
C9	IL2RA	PR, TAC, MMF	+	aCNIT
C10	IL2RA	PR, CSA, MMF	+	cCNIT
C11	IL2RA	PR, TAC, MMF	+	aCNIT
C12	IL2RA	PR, TAC, MMF	+	aCNIT
I13	IL2RA	PR, TAC, MMF	−	IFTA (G2)
I14	IL2RA	PR, TAC, MMF	−	IFTA (G1)
I15	IL2RA	PR, TAC, MMF	−	IFTA (G2)
I16	IL2RA	PR, TAC, MMF	−	IFTA (G2)
I17	IL2RA	PR, CSA	−	IFTA (G2)

IS, immunosuppressive treatment; CNI, calcineurin inhibitors; N, NKF, C, CNIT; I, IFTA; IL2RA, interleukin 2 receptor antagonists; rATG, rabbit anti-thymocyte globulin; PR, prednisone; CSA, cyclosporine A; TAC, tacrolimus; MMF, mycophenolate mofetil; NKF, normal kidney function; aCNIT, acute CNI toxicity; cCNIT, chronic CNI toxicity; IFTA (G), interstitial fibrosis and tubular atrophy (grade).

## Supplementary Materials

Supplementary materials can be found at https://www.mdpi.com/1422-0067/21/20/7569/s1.

## Abbreviations

aCNIT	acute calcineurin inhibitors toxicity
ANOVA	Analysis of variance
BP	Biological Process
CC	Cellular Component
cCNIT	chronic calcineurin inhibitors toxicity
CD	cluster of differentiation
CNI	calcineurin inhibitors
CNIT	calcineurin inhibitors toxicity
CSA	cyclosporine A
DM	diabetes mellitus type 2
ES	enrichment score
F	female
FDR	false discovery rate
G	grade
GO	Gene Ontology
GSEA	Gene Set Enrichment Analysis
HT	arterial hypertension
iBAQ	Intensity-based absolute quantification
IFTA	interstitial fibrosis and tubular atrophy
IL	interleukin
IL	interleukin
IL2RA	interleukin 2 receptor antagonists
IS	immunosuppressive treatment
LC-MS/MS	liquid chromatography tandem mass-spectrometry
LD	living donor
M	male
MFI	mean fluorescence intensity
MMF	mycophenolate mofetil
MSigDB	Molecular Signatures Database
NES	normalized enrichment score
NKF	normal kidney function
PBS	phosphate-buffered saline
PCA	Principal Component Analysis
PR	prednisone
rATG	rabbit anti-thymocyte globulin
RT	Renal transplantation
SEC	size-exclusion chromatography
TAC	tacrolimus
uEV	Urinary Extracellular Vesicles
