# Supplementary material for: Proteomic Characterization of Urinary Extracellular Vesicles from Kidney-Transplanted Patients Treated with Calcineurin Inhibitors
Source: Int J Mol Sci. 2020 Oct 14;21(20):7569. doi: 10.3390/ijms21207569 (PMC7589460; doi:10.3390/ijms21207569)
Supplement: Supplementary file 1 [file ijms-21-07569-s001.zip › Supplementary material IJMS_Rev1.docx]

Supplementary Figure 1. Representative histological photographs of kidney allograft biopsies showing different alterations. (**a**) Hematoxilin/eosin stained biopsy with minimal histological alterations (100x, scale bar represents 100 μm). (**b**) Hematoxilin/eosin stained biopsy showing acute tubular CNIT with isometric vacuolization of the proximal tubular epithelial cells’ cytoplasm (indicated with arrows) (200x, scale bar represents 50 μm). (**c**) Periodic acid-Schiff staining of a kidney with chronic vascular CNIT showing severe arteriolar hyalinosis with circumferential involvement. Arrows indicate adventitial hyaline deposits (pearl-like pattern) (Banff score ah3) (400x, scale bar represents 20 μm). (**d**-**e**) Silver methenamine (Jones)-stained biopsies with IFTA grades 1 and 2, respectively. Asterisks indicate scarred areas of cortex affected by inflammation (20x, scale bars represent 500 μm).

Supplementary table 1. Banff scoring results of the histopathological analysis of kidney biopsies from patients of the discovery cohort.

| **Group, sample** | **i** | **t** | **v** | **g** | **ah** | **ci** | **ct** | **cv** | **cg** | **mm** | **ptc** | **ti** | **c4d** | **iatr** |
| --- | --- | --- | --- | --- | --- | --- | --- | --- | --- | --- | --- | --- | --- | --- |
| C8 | 0 | 0 | 0 | 0 | 0 | 0 | 0 | 2 | 0 | 0 | 0 | 1 | 0 | 1 |
| C9 | 0 | 0 | 0 | 0 | 0 | 1 | 1 | 0 | 0 | 0 | 0 | 1 | 0 | 1 |
| C10 | 0 | 0 | 0 | 0 | 3 | 2 | 2 | 1 | 0 | 0 | 0 | 3 | 0 | 3 |
| C11 | 0 | 0 | 0 | 0 | 0 | 1 | 1 | 1 | 0 | 1 | 0 | 0 | 0 | 0 |
| C12 | 0 | 0 | 0 | 0 | 0 | 0 | 0 | 1 | 0 | 0 | 0 | 0 | 0 | 0 |
| I13 | 0 | 0 | 0 | 0 | 0 | 1 | 1 | 1 | 0 | 0 | 0 | 2 | 0 | 2 |
| I14 | 0 | 0 | 0 | 0 | 3 | 1 | 1 | 1 | 0 | 0 | 0 | 1 | 0 | 1 |
| I15 | 0 | 0 | 0 | 1 | 1 | 1 | 1 | 0 | 0 | 1 | 0 | 1 | 0 | 1 |
| I16 | 0 | 0 | 0 | 0 | 3 | 2 | 2 | 3 | 0 | 1 | 0 | 1 | 0 | 1 |
| I17 | 0 | 0 | 0 | 0 | 3 | 2 | 2 | 1 | 0 | 1 | 0 | 1 | 0 | 1 |

i, interstitial infiltrate; t, tubulitis; v, vasculitis; g, glomerulitis; ah, arteriolar hyalinosis; ci, chronic interstitial lesions; ct, chronic tubular lesions; cv, chronic vascular lesions; cg, chronic glomerular lesions; mm, mesangial matrix increase; ptc, peritubular capillaritis; ti, total interstitial inflammation; c4d, C4d deposition; iatr, inflammation in areas of tubular atrophy.

Supplementary table 2. Gene Ontology – Biological Process most significantly enriched terms in the 730 proteins found in the mass-spectrometry analysis.

| **Term description** | **Observed gene count** | **Background gene count** | **FDR** |
| --- | --- | --- | --- |
| Vesicle-mediated transport | 261 | 1699 | 3.12e-91 |
| Regulated exocytosis | 178 | 691 | 4.79e-89 |
| Exocytosis | 184 | 774 | 1.53e-87 |
| Secretion by cell | 192 | 959 | 1.05e-80 |
| Secretion | 198 | 1070 | 2.14e-78 |
| Leukocyte mediated immunity | 156 | 632 | 4.94e-75 |
| Neutrophil mediated immunity | 140 | 498 | 7.63e-73 |
| Neutrophil activation involved in immune response | 138 | 489 | 6.03e-72 |
| Myeloid leukocyte mediated immunity | 141 | 519 | 7.00e-72 |
| Neutrophil degranulation | 137 | 485 | 1.61e-71 |
| Leukocyte degranulation | 139 | 507 | 2.53e-71 |
| Myeloid cell activation involved in immune response | 140 | 519 | 3.81e-71 |
| Myeloid leukocyte activation | 144 | 574 | 7.13e-70 |
| Immune effector process | 174 | 927 | 6.09e-69 |
| Transport | 355 | 4130 | 2.66e-67 |
| Cell activation involved in immune response | 144 | 620 | 3.51e-66 |
| Establishment of localization | 358 | 4248 | 5.63e-66 |
| Leukocyte activation involved in immune response | 143 | 616 | 1.06e-65 |
| Cell activation | 173 | 1024 | 1.66e-62 |
| Localization | 392 | 5233 | 2.37e-61 |

Supplementary table 3. Gene Ontology – Cellular Component most significantly enriched terms in the 730 proteins found in the mass-spectrometry analysis.

| **Term description** | **Observed gene count** | **Background gene count** | **FDR** |
| --- | --- | --- | --- |
| Vesicle | 311 | 2318 | 2.46e-100 |
| Extracellular region | 312 | 2505 | 3.40e-93 |
| Cytoplasmic vesicle | 291 | 2226 | 5.22e-90 |
| Secretory granule | 190 | 828 | 1.23e-89 |
| Cytoplasmic vesicle part | 234 | 1447 | 1.44e-85 |
| Secretory vesicle | 195 | 948 | 3.94e-85 |
| Cytoplasmic vesicle lumen | 115 | 340 | 5.28e-67 |
| Secretory granule lumen | 111 | 323 | 3.13e-65 |
| Extracellular region part | 197 | 1375 | 2.27e-62 |
| Endomembrane system | 349 | 4347 | 4.70e-59 |
| Extracellular space | 175 | 1134 | 8.46e-59 |
| Cytoplasmic part | 524 | 9377 | 4.96e-54 |
| Cytoplasm | 560 | 11238 | 7.03e-44 |
| Cell periphery | 355 | 5254 | 1.07e-42 |
| Plasma membrane | 347 | 5159 | 8.21e-41 |
| Lysosome | 106 | 582 | 8.55e-40 |
| Vacuole | 112 | 682 | 1.92e-38 |
| Whole membrane | 166 | 1554 | 2.56e-36 |
| Vacuolar lumen | 61 | 172 | 6.27e-36 |
| Azurophil granule | 58 | 155 | 3.53e-35 |

Supplementary table 4. List of the proteins significantly differently expressed shown in the volcano plot of CNIT vs. NKF (Figure 3b).

| **Gene name** | **Fold change** | **-log(*p*-value)** |
| --- | --- | --- |
| CTSZ | 17.09682 | 12.33218 |
| RAB8A | 15.14346 | 11.75521 |
| SERPINC1 | 16.64265 | 8.38317 |
| NAPSA | 16.46061 | 4.400914 |
| TTR | 18.90059 | 4.059741 |
| GSTP1 | 16.6204 | 3.910614 |
| YWHAQ | 14.32559 | 3.870245 |
| CAPG | 16.03821 | 3.866636 |
| FGA | 15.49457 | 3.755747 |
| GNAI1 | 14.04115 | 3.753342 |
| PTGR1 | 14.11435 | 3.673125 |
| STXBP2 | 13.22334 | 3.640962 |
| CNP | 13.96733 | 3.619861 |
| RBP4 | 18.42533 | 3.606263 |
| SLC3A2 | 13.04238 | 3.57344 |
| CA1 | 16.96889 | 3.483952 |
| ADIRF | 16.96444 | 3.473245 |
| CAPZA1 | 13.09425 | 3.159562 |
| EIF5A2 | 14.04444 | 3.157831 |
| CRABP2 | 14.17305 | 3.153388 |
| UPK1B | 14.80527 | 3.151623 |
| TUBB | 14.20602 | 3.147056 |
| CBR1 | 13.52186 | 3.140921 |
| CTSD | 14.12 | 3.129715 |
| FBP1 | 13.27951 | 3.11967 |
| RAB1B | 12.49896 | 3.11859 |
| FLOT1 | 13.09407 | 3.11681 |
| RAB8B | 11.55652 | 3.106566 |
| CAPN5 | 13.2108 | 3.091306 |
| GNA13 | 12.91073 | 3.089945 |
| ARHGDIB | 14.245 | 3.083094 |
| DSTN | 13.25582 | 3.074183 |
| PTPN13 | 12.15206 | 3.070595 |
| PLSCR1 | 14.0724 | 3.06606 |
| RAP1A | 13.99524 | 3.059056 |
| BAIAP2L1 | 12.53215 | 3.050912 |
| EPS8 | 14.28895 | 3.04115 |
| CST3 | 16.441 | 3.021803 |
| SLC34A2 | 13.1171 | 3.016683 |
| IDH1 | 13.63968 | 3.001035 |
| PPL | 11.21278 | 2.922073 |
| PKM | 13.76167 | 2.77458 |
| HSPG2 | 12.35273 | 2.769925 |
| HSP90AA1 | 13.65436 | 2.73587 |
| ACTN4 | 13.33733 | 2.727924 |
| EPS8L2 | 14.65043 | 2.710358 |
| PGK1 | 13.20971 | 2.676922 |
| EFEMP1 | 13.67455 | 2.644454 |
| VPS37B | 12.34922 | 2.578884 |
| TPI1 | 14.26845 | 2.563448 |
| PRDX6 | 13.34694 | 2.562729 |
| ASAH1 | 12.58008 | 2.409175 |
| EHD1 | 12.88952 | 2.345764 |
| LMAN2 | 13.51137 | 2.339659 |
| YWHAG | 12.26183 | 2.272777 |
| CALB1 | 12.37555 | 2.209791 |
| ANXA6 | 11.45126 | 2.165348 |
| AGT | 13.93267 | 2.164472 |
| DPP4 | 12.11966 | 2.163453 |
| ITIH4 | 12.73345 | 2.159698 |
| ASS1 | 12.16517 | 2.152552 |
| PGAM1 | 13.19666 | 2.124046 |
| MYH9 | 10.01056 | 2.117177 |
| MARCKS | 11.63666 | 2.115895 |
| CA2 | 12.35211 | 2.061382 |
| PRDX1 | 12.44137 | 2.051854 |
| SNAP23 | 12.01325 | 2.035146 |
| RAB7A | 10.90947 | 2.022379 |
| RHOA | 12.33847 | 2.016213 |
| MSN | 12.20578 | 2.010916 |
| AKR1A1 | 11.45235 | 2.003496 |

Supplementary table 5. List of the proteins significantly differently expressed shown in the volcano plot of CNIT vs. IFTA (Figure 3c). A negative fold change indicates more expression in the IFTA group.

| **Gene name** | **Fold Change** | **-log(*p*-value)** |
| --- | --- | --- |
| ADIRF | 19.57731 | 8.591249 |
| CAPG | 18.2191 | 8.235004 |
| STXBP2 | 15.14143 | 7.948807 |
| GNAI1 | 15.98835 | 7.419415 |
| ATP1A1 | 15.60777 | 7.307434 |
| PPL | 12.02056 | 2.922208 |
| VPS37B | 13.47743 | 2.781843 |
| SNCG | 14.83107 | 2.723545 |
| RAB8A | 12.38739 | 2.662239 |
| TSG101 | 14.43177 | 2.572905 |
| EHD4 | 15.24505 | 2.515646 |
| VPS28 | 15.43811 | 2.490042 |
| ANXA4 | 14.45527 | 2.440012 |
| CAPZA1 | 13.09425 | 2.402834 |
| UPK2 | 15.1833 | 2.401713 |
| CRABP2 | 14.17305 | 2.398072 |
| CD47 | 13.33802 | 2.39788 |
| UPK1A | 14.25236 | 2.39681 |
| UPK1B | 14.80527 | 2.396711 |
| ANXA6 | 13.10347 | 2.388609 |
| CD2AP | 12.67408 | 2.375768 |
| RAB1B | 12.49896 | 2.37124 |
| FLOT1 | 13.09407 | 2.369867 |
| RAB8B | 11.55652 | 2.361971 |
| EVPL | 12.62046 | 2.358555 |
| PPP2R1A | 12.01373 | 2.358262 |
| CAPN5 | 13.2108 | 2.350211 |
| THY1 | 15.95974 | 2.346988 |
| VPS4A | 13.51522 | 2.343438 |
| S100A11 | 15.07003 | 2.337983 |
| DSTN | 13.25582 | 2.337018 |
| CALB1 | 13.51569 | 2.336079 |
| FABP5 | 14.20961 | 2.333802 |
| CLIC6 | 13.24979 | 2.330843 |
| MYO1C | 12.86527 | 2.315403 |
| ANXA7 | 12.63252 | 2.313072 |
| SLC34A2 | 13.1171 | 2.292739 |
| RHOA | 15.02891 | 2.192228 |
| DPP4 | 14.22212 | 2.1078 |
| HIST1H4A | -17.75791 | 2.951101 |
| IGHV4-28 | -13.0448 | 2.379408 |
| HRG | -16.79686 | 2.64356 |

Supplementary table 6. Top 20 most enriched Gene Ontology - Biological Process terms in CNIT compared to NKF from GSEA.

| **Gene ontology – Biological process term** | **SIZE** | **NES** | **NOM p-val** | **FDR q-val** |
| --- | --- | --- | --- | --- |
| Regulation of protein serine threonine kinase activity | 34 | 1.515 | 0.019 | 0.542 |
| Cellular component morphogenesis | 47 | 1.520 | 0.007 | 0.548 |
| Regulation of peptide transport | 17 | 1.489 | 0.035 | 0.567 |
| Regulation of organelle organization | 82 | 1.483 | 0.005 | 0.569 |
| Golgi vesicle transport | 22 | 1.478 | 0.028 | 0.571 |
| Regulation of actin filament length | 26 | 1.559 | 0.014 | 0.572 |
| Response to nutrient | 17 | 1.473 | 0.049 | 0.573 |
| Localization within membrane | 15 | 1.521 | 0.030 | 0.582 |
| Regulation of hormone levels | 33 | 1.567 | 0.008 | 0.582 |
| Positive regulation of cellular component biogenesis | 39 | 1.527 | 0.015 | 0.582 |
| Small gtpase mediated signal transduction | 38 | 1.490 | 0.014 | 0.588 |
| Cell cycle phase transition | 15 | 1.652 | 0.008 | 0.588 |
| Cell projection assembly | 20 | 1.495 | 0.026 | 0.592 |
| Regulation of ras protein signal transduction | 17 | 1.538 | 0.024 | 0.596 |
| Positive regulation of protein serine threonine kinase activity | 19 | 1.371 | 0.096 | 0.601 |
| Actin filament organization | 29 | 1.498 | 0.022 | 0.602 |
| Neuron projection morphogenesis | 25 | 1.545 | 0.012 | 0.603 |
| Negative regulation of response to wounding | 24 | 1.368 | 0.079 | 0.605 |
| Gliogenesis | 16 | 1.372 | 0.080 | 0.607 |
| Regulation of blood pressure | 16 | 1.528 | 0.038 | 0.614 |

NES, Normalized enrichment score; NOM p-val, nominal *p*-value; FDR q-val, false discovery rate *q*-value.

Supplementary table 7. Enriched Gene Ontology - Biological Process terms in NKF compared to CNIT from GSEA.

| **Gene ontology – Biological process term** | **SIZE** | **NES** | **NOM p-val** | **FDR q-val** |
| --- | --- | --- | --- | --- |
| Negative regulation of immune response | 16 | -1.224 | 0.174 | 0.871 |
| Aminoglycan metabolic process | 17 | -0.631 | 0.951 | 0.966 |
| Response to radiation | 16 | -1.26 | 0.162 | 1 |
| B cell mediated immunity | 18 | -0.741 | 0.8 | 1 |
| Carbohydrate derivative catabolic process | 17 | -0.814 | 0.806 | 1 |
| Cellular response to cytokine stimulus | 19 | -0.843 | 0.821 | 1 |
| Complement activation | 21 | -0.785 | 0.84 | 1 |
| Adaptive immune response based on somatic recombination of immune receptors built from immunoglobulin superfamily domains | 19 | -0.772 | 0.853 | 1 |
| Humoral immune response mediated by circulating immunoglobulin | 18 | -0.803 | 0.906 | 1 |
| Ammonium ion metabolic process | 15 | -0.676 | 0.914 | 1 |

NES, Normalized enrichment score; NOM p-val, nominal *p*-value; FDR q-val, false discovery rate *q*-value.

Supplementary table 8. Top 20 most enriched Gene Ontology - Biological Process terms in CNIT compared to IFTA from GSEA.

| **Gene ontology – Biological process term** | **Size** | **NES** | **NOM p-val** | **FDR q-val** |
| --- | --- | --- | --- | --- |
| Regulation of actin filament length | 29 | 1.793 | 0.001 | 0.058 |
| Epithelial cell differentiation | 59 | 1.798 | 0.000 | 0.068 |
| Regulation of protein complex disassembly | 29 | 1.801 | 0.000 | 0.083 |
| Cell cycle | 57 | 1.757 | 0.001 | 0.087 |
| Actin filament organization | 32 | 1.641 | 0.009 | 0.091 |
| Regulation of actin filament based process | 47 | 1.712 | 0.000 | 0.092 |
| Regulation of cell cycle process | 28 | 1.642 | 0.002 | 0.093 |
| Modulation of synaptic transmission | 25 | 1.644 | 0.013 | 0.095 |
| Vesicle organization | 41 | 1.741 | 0.001 | 0.096 |
| Intracellular protein transport | 61 | 1.645 | 0.002 | 0.097 |
| Multivesicular body organization | 20 | 1.633 | 0.007 | 0.098 |
| Regulation of cell cycle | 38 | 1.630 | 0.007 | 0.098 |
| Endomembrane system organization | 54 | 1.714 | 0.000 | 0.098 |
| Regulation of cellular component biogenesis | 80 | 1.611 | 0.001 | 0.099 |
| Regulation of protein polymerization | 27 | 1.655 | 0.003 | 0.099 |
| Regulation of cellular component size | 46 | 1.647 | 0.002 | 0.099 |
| Membrane budding | 27 | 1.694 | 0.002 | 0.099 |
| Multi organism membrane organization | 19 | 1.650 | 0.014 | 0.100 |
| Negative regulation of protein complex disassembly | 17 | 1.683 | 0.009 | 0.101 |
| Virion assembly | 22 | 1.612 | 0.012 | 0.101 |

NES, Normalized enrichment score; NOM p-val, nominal *p*-value; FDR q-val, false discovery rate *q*-value.

Supplementary table 9. Top 20 most enriched Gene Ontology - Biological Process terms in IFTA compared to CNIT from GSEA.

| **Gene ontology – Biological process term** | **Size** | **NES** | **NOM p-val** | **FDR q-val** |
| --- | --- | --- | --- | --- |
| Protein activation cascade | 51 | -2.909 | 0.000 | 0.000 |
| Complement activation | 37 | -2.852 | 0.000 | 0.000 |
| Humoral immune response mediated  By circulating immunoglobulin | 32 | -2.754 | 0.000 | 0.000 |
| Humoral immune response | 51 | -2.752 | 0.000 | 0.000 |
| B cell mediated immunity | 32 | -2.682 | 0.000 | 0.000 |
| Adaptive immune response based on  somatic recombination of immune receptors  built from immunoglobulin superfamily domains | 35 | -2.671 | 0.000 | 0.000 |
| Lymphocyte mediated immunity | 39 | -2.491 | 0.000 | 0.000 |
| Aminoglycan metabolic process | 23 | -2.448 | 0.000 | 0.000 |
| Mucopolysaccharide metabolic process | 15 | -2.416 | 0.000 | 0.000 |
| Regulation of protein maturation | 25 | -2.367 | 0.000 | 0.001 |
| Regulation of acute inflammatory response | 23 | -2.315 | 0.000 | 0.001 |
| Regulation of humoral immune response | 21 | -2.316 | 0.000 | 0.001 |
| Regulation of protein activation cascade | 22 | -2.280 | 0.000 | 0.002 |
| Carbohydrate derivative catabolic process | 26 | -2.171 | 0.000 | 0.003 |
| Negative regulation of coagulation | 17 | -2.142 | 0.000 | 0.004 |
| Negative regulation of wound healing | 17 | -2.122 | 0.000 | 0.005 |
| Adaptive immune response | 42 | -2.110 | 0.000 | 0.005 |
| Defense response to bacterium | 36 | -2.086 | 0.000 | 0.005 |
| Platelet degranulation | 47 | -2.070 | 0.000 | 0.006 |
| Blood coagulation fibrin clot formation | 15 | -2.055 | 0.006 | 0.006 |

NES, Normalized enrichment score; NOM p-val, nominal *p*-value; FDR q-val, false discovery rate *q*-value.
