# Supplementary figures and images for: Proteomic Characterization of Urinary Extracellular Vesicles from Kidney-Transplanted Patients Treated with Calcineurin Inhibitors
Source: Int J Mol Sci. 2020 Oct 14;21(20):7569. doi: 10.3390/ijms21207569 (PMC7589460; doi:10.3390/ijms21207569)

Fig S1

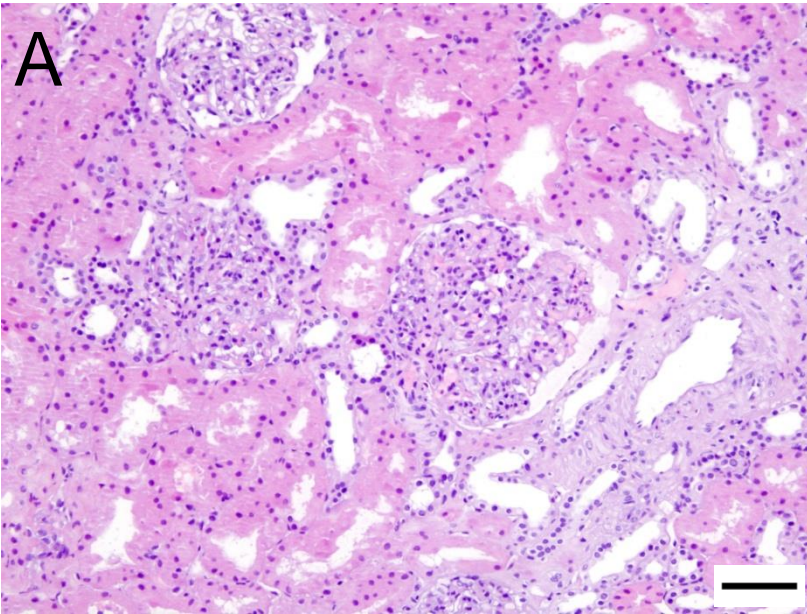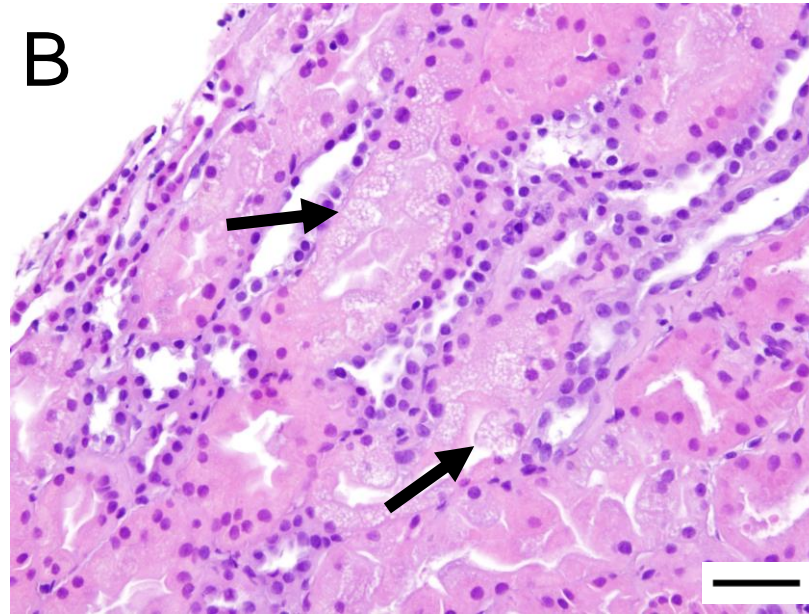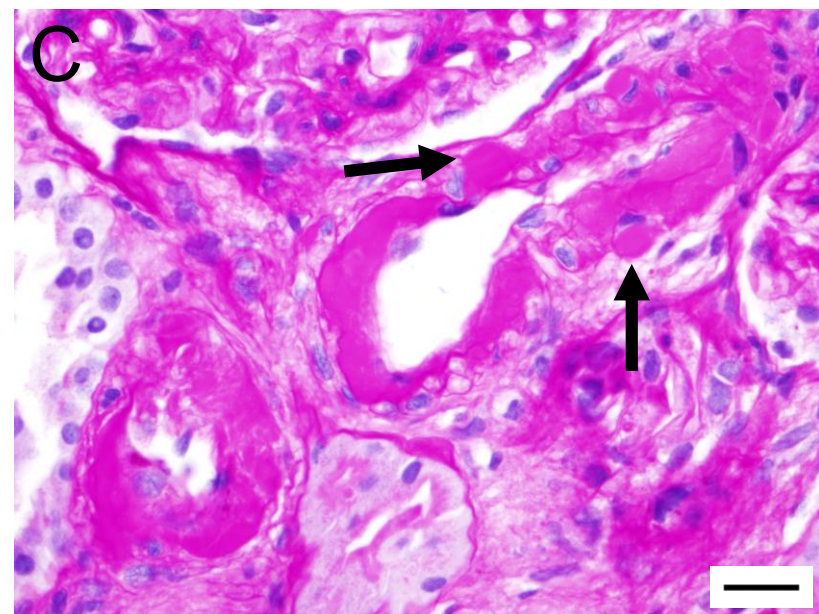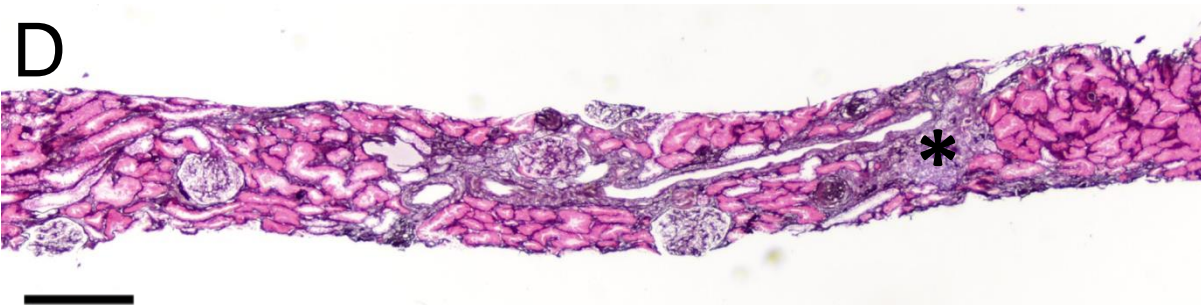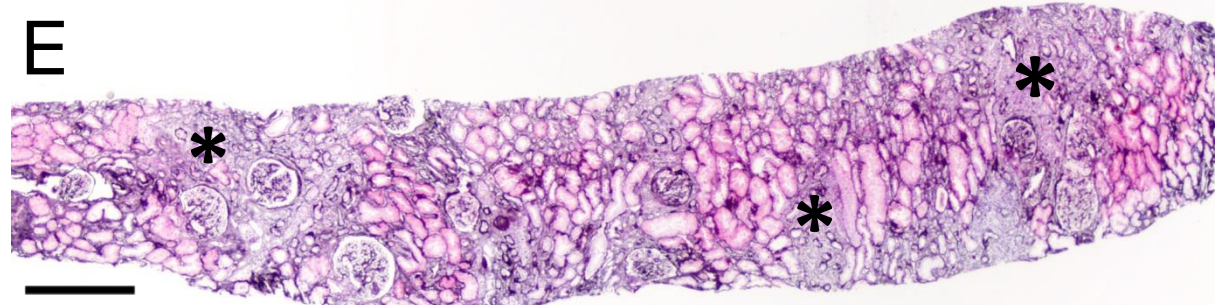

Supplement: Supplementary file 1 [file ijms-21-07569-s001.zip › FigS1_Rev1.pdf]
